# Comprehensive Analysis of E3 Ubiquitin Ligases Reveals Ring Finger Protein 223 as a Novel Oncogene Activated by KLF4 in Pancreatic Cancer

**DOI:** 10.3389/fcell.2021.738709

**Published:** 2021-10-14

**Authors:** Lei Feng, Jieqing Wang, Jianmin Zhang, Jingfang Diao, Longguang He, Chaoyi Fu, Hui Liao, Xiaoping Xu, Yi Gao, Chenjie Zhou

**Affiliations:** ^1^Department of Hepatobiliary Surgery II, Guangdong Provincial Research Center for Artificial Organ and Tissue Engineering, Guangzhou Clinical Research and Transformation Center for Artificial Liver, Institute of Regenerative Medicine, Zhujiang Hospital, Southern Medical University, Guangzhou, China; ^2^The First Affiliated Hospital, Sun Yat-sen university, Guangzhou, China; ^3^Guangdong Provincial Hospital of Traditional Chinese Medicine, Guangzhou, China; ^4^Gaozhou People’s Hospital, Gaozhou, China; ^5^State Key Laboratory of Organ Failure Research, Southern Medical University, Guangzhou, China

**Keywords:** pancreatic cancer, E3 ubiquitin ligase, prognosis, RNF223, KLF4, metabolism

## Abstract

Pancreatic cancer is one of the major malignancies and causes of mortality worldwide. E3 ubiquitin–protein ligases transfer activated ubiquitin from ubiquitin-conjugating enzymes to protein substrates and confer substrate specificity in cancer. In this study, we first downloaded data from The Cancer Genome Atlas pancreatic adenocarcinoma dataset, acquired all 27 differentially expressed genes (DEGs), and identified genomic alterations. Then, the prognostic significance of DEGs was analyzed, and eight DEGs (MECOM, CBLC, MARCHF4, RNF166, TRIM46, LONRF3, RNF39, and RNF223) and two clinical parameters (pathological N stage and T stage) exhibited prognostic significance. RNF223 showed independent significance as an unfavorable prognostic marker and was chosen for subsequent analysis. Next, the function of RNF223 in the pancreatic cancer cell lines ASPC-1 and PANC-1 was investigated, and RNF223 silencing promoted pancreatic cancer growth and migration. To explore the potential targets and pathways of RNF223 in pancreatic cancer, quantitative proteomics was applied to analyze differentially expressed proteins, and metabolism-related pathways were primarily enriched. Finally, the reason for the elevated expression of RNF223 was analyzed, and KLF4 was shown to contribute to the increased expression of RNF233. In conclusion, this study comprehensively analyzed the clinical significance of E3 ligases. Functional assays revealed that RNF223 promotes cancer by regulating cell metabolism. Finally, the elevated expression of RNF223 was attributed to KLF4-mediated transcriptional activation. This study broadens our knowledge regarding E3 ubiquitin ligases and signal transduction and provides novel markers and therapeutic targets in pancreatic cancer.

## Introduction

Pancreatic cancer (PC) is one of the major malignancies and causes of mortality worldwide (Cheng et al., 2019; Mizrahi et al., 2020; Siegel et al., 2020). The most common type of PC is adenocarcinoma, accounting for 95% of cases, which is classified as pancreatic ductal adenocarcinoma (He et al., 2014; Martens et al., 2019). The prognosis for PC remains poor, with only 4.4% of patients reaching a 5-year survival rate (Mizrahi et al., 2020). Risk factors for developing PC include family history, obesity, type 2 diabetes, and tobacco use (Mizrahi et al., 2020). Diagnosis of PC often occurs at a late stage, meaning that more than 80% of patients with PC are unsuitable for surgical resection (Buscail et al., 2020). Chemotherapy, targeted therapy, and immunotherapy are now the most widely used treatments for PC (Bear et al., 2020; Christenson et al., 2020; Hosein et al., 2020). However, because of delayed disease detection and the limited efficacy of systemic therapies, the prognosis for this disease remains very poor (Grossberg et al., 2020). Therefore, insights regarding the regulatory mechanisms underlying PC progression are required to identify novel diagnostic and/or prognostic markers.

Ubiquitination plays an essential role in protein posttranslational modification and is strongly linked to various diseases (Rape, 2018). E3 ubiquitin–protein ligases transfer activated ubiquitin from ubiquitin-conjugating enzymes to protein substrates and confer substrate specificity in cancer (Zheng and Shabek, 2017; Senft et al., 2018). The RING finger (RNF) protein family is a complex set of proteins containing an RNF domain with more than 200 members having been identified (Joazeiro and Weissman, 2000; Fang et al., 2003; Lipkowitz and Weissman, 2011). Many RNF family members have been reported to play key roles in carcinogenesis (Lipkowitz and Weissman, 2011), such as RNF45 (Tsai et al., 2007), RNF6 (Liang et al., 2018), RNF4 (Plechanovová et al., 2011), RNF7 (Sun and Li, 2013), RNF168 (Devgan et al., 2011), RNF183 (Geng et al., 2017), RNF20 (Dickson et al., 2016), and RNF180 (Deng et al., 2016). In PC, RNF13 is involved in tumorigenesis (Zhang et al., 2009). As a biomarker candidate of PC, RNF6 facilitates PC metastasis by enhancing the c-Myc–mediated Warburg effect (Qiu et al., 2021). RNF43 mutation might cause downregulation of the expression of ring finger protein 43 and synergistically associates with GNAS mutations during the development of PC (Sakamoto et al., 2015). In addition, mutational inactivation of RNF43 confers Wnt dependency and could be used as a predictive biomarker for the clinical development of Wnt inhibitors in PC (Jiang et al., 2013). Identification of additional RNF family members associated with PC will help to elucidate the process of carcinogenesis and to develop new therapeutic strategies.

In this study, we first downloaded gene expression data from The Cancer Genome Atlas (TCGA) pancreatic adenocarcinoma (PAAD) dataset and analyzed the expression differences in E3 ubiquitin ligases (Medvar et al., 2016). Then, the prognostic significance of differentially expressed genes (DEGs) was analyzed. Of the DEGs, RNF223 showed independent significance as an unfavorable prognostic marker and was chosen for subsequent analysis. Next, the function of RNF223 in the PC cell lines ASPC-1 and PANC-1 was investigated using shRNA-mediated RNA silencing. To explore the potential targets and pathways of RNF223 in PC, quantitative proteomics was applied to analyze differentially expressed proteins (DEPs) and their functions in RNF223-silenced ASPC-1 cells. Finally, the mechanism for the elevated expression of RNF223 in PC was analyzed, and the regulatory mechanism was validated using a luciferase assay. The flowchart was shown in Supplementary Figure 1. This study deepens our understanding of the clinical significance and role of the E3 ligase RNF223 in pancreatic cancer.

## Materials and Methods

### The Cancer Genome Atlas Pancreatic Adenocarcinoma Dataset and E3 Ligase Acquisition

TCGA PAAD transcriptome FPKM data were downloaded from the GDC Data Portal^1^. Clinical data, such as age, sex, clinical stage, and survival time, were also downloaded. The 377 E3 ligase genes were acquired from the online database^2^.

### Differentially Expressed Genes and Functional Analysis

Gene expression in PC and control samples was compared using the LIMMA R package. A fold change ≥ 2 or ≤ 0.5 and *p* < 0.05 were set as the cutoff values for EG screening. The heatmap R package was used to draw heatmaps. The Kyoto Encyclopedia of Genes and Genomes (KEGG) pathway analysis, Gene Ontology (GO) enrichment analysis, and protein–protein interaction enrichment analysis for DEGs were also performed using the EnrichR (Kuleshov et al., 2016) website tool^3^.

### Genomic Alterations in Pancreatic Adenocarcinoma Samples

The genomic alteration (GA) information of DEGs was acquired using cBioPortal^4^. Two PAAD datasets were included in this study, the TCGA Pan-cancer atlas and the UTSW study (Witkiewicz et al., 2015). All 184 and 109 samples from the two datasets were included.

### Cell Culture and Gene Silencing

The PANC-1 and ASPC-1 human PC cell lines were obtained from the American Type Culture Collection (United States) and cultured at 37°C with 95% air and 5% CO_2_. PANC-1 and ASPC-1 cells were cultured in Dulbecco modified Eagle medium (Gibco, Germany) supplemented with 10% fetal calf serum (Germany), 2 mM L-glutamine, 105 U/L penicillin, and 100 mg/L streptomycin. Short hairpin RNAs specifically targeting RNF223 and Kruppel-like factor 4 (KLF4) were designed and synthesized by Generay Biotech (Shanghai) Co., Ltd., RNF223 shRNAs were as follows: shRNA1 (5′–3′): GCACAGCAGCCACTGGAAGTC, shRNA2 (5′–3′): GCGAAAGGAGCCTGGCATCTC, and shRNA3 (5′–3′): GGAGCCTGGCATCTCTGAGGA.

KLF4 shRNAs were as follows: shRNA1 (5′–3′): GCTCC ATTACCAAGAGCTCAT, shRNA2 (5′–3′): CCAGCCAGAAA GCACTACAAT, and shRNA3 (5′–3′): GCCTTACACATGAAG AGGCAT.

### Cell Counting Kit-8 Assay

The cell proliferation reagent WST-8 (Roche, Germany) was measured. Cell growth: 10 μL of cell counting kit-8 (CCK8) was added to each well at the time of harvest after plating cells in 96-well microtiter plates (Corning, NY) at 1.0 × 10^3^/well, according to the manufacturer’s instructions. Cellular viability was determined by measuring the absorbance of the converted dye at 450 nm 2 h after adding CCK8.

### Wound Healing Assay

PANC-1 and ASPC-1 cells were seeded into 6-well plates and incubated for 24 h, and a linear wound was created by dragging a 100-μL pipette tip through the monolayer prior to transfection. Cellular debris was removed by gentle washes with culture medium, following which transfection was immediately performed, and the cells were allowed to migrate for an additional 48 h. The healing process was dynamically imaged after the wound was introduced using a microscope (Olympus 600 Autobiochemical Analyzer, Tokyo, Japan). Migration distance was measured using images (five fields) taken at each indicated time point. The gap size was analyzed using Image Pro Plus 6.0 software. The residual gap between the migrating cells from the opposing wound edge is expressed as a percentage of the initial gap size.

### Quantitative Real-Time Polymerase Chain Reaction

Total RNA was extracted from ASPC-1 and PANC-1 cells using TRIzol^®^ RNA Isolation Reagent (Invitrogen, Carlsbad, CA) according to the manufacturer’s instructions. Reverse transcription was performed using the PrimeScript^TM^ RT reagent kit (Takara, Dalian, China). All mRNA levels were normalized to the housekeeping gene GAPDH. The following RNF223 and KLF4 primers were used in this study: RNF223: 5′-TGATGCTCTTCTGTGTGGCA-3′ (F) and 5′-TTATCAGTCAG AGGCCCGAG-3′ (R). KLF4: 5′-CCCACATGAAGCGACTTC CC-3′ (F) and 5′-CAGGTCCAGGAGATCGTTGAA-3′(R). The GAPDH primers were as follows: 5′-TGACTTCAACAGC GACACCCA-3′ (F) and 5′- CACCCTGTTGCTGTAGCCAAA-3′ (R). All samples were treated under the same conditions and analyzed by quantitative reverse transcriptase–polymerase chain reaction (qRT-PCR) using SYBR Premix ExTaq^TM^ (Takara, Dalian China) according to the manufacturer’s protocol.

### Prognostic Significance of Genes

Univariate Cox regression analysis was performed to identify prognosis-associated E3 ligase genes. Genes with a hazard ratio (HR) < 1 were considered favorable for OS, whereas HR > 1 presented unfavorable for OS. Genes with *p* < 0.05 were considered significant markers. The Kaplan–Meier survival plot was constructed using the “survplot” R package. Pan-cancer survival analysis was conducted using the KM plotter online tool (Nagy et al., 2021)^5^.

### Data-Independent Acquisition Quantitative Proteomics

Data-independent acquisition quantitative proteomics was conducted according to a previous study (Zhang et al., 2021). Briefly, cells underwent protein extraction and trypsin digestion into peptides, and then a spectral library was generated and quantified. Ten fractions were collected, and each fraction was dried in a vacuum concentrator. The fractions were redissolved in 0.1% formic acid and analyzed using nanospray liquid chromatography with tandem mass spectrometry (LC-MS/MS) on an Orbitrap Fusion Lumos Tribrid (Thermo Fisher Scientific, MA, United States) coupled to a Waters nanoACQUITY UPLC System (Waters, MA, United States). The mass spectrometer was run in DDA mode and automatically switched between MS and MS/MS modes. The DDA data were processed and analyzed using Spectronaut X (Biognosys, Schlieren, Switzerland) with default settings to generate an initial target list. A false discovery rate cutoff at the precursor and protein levels was applied at 1%. Finally, proteins were identified and quantified.

### ChIP Sequencing Analysis of the Transcription Factors in RNF223

The ChIP sequencing peaks of RNF223 transcription factors were identified using the online tool ChIP-Atlas^6^. PC, including cell lines, datasets in bigwig format were downloaded and imported into the IGV browser. Transcription factors with binding peaks within the distance of ≤ 1 kb from the transcription start sites were considered candidate peaks.

### Luciferase Reporter Assay

A luciferase reporter assay was performed according to a standard protocol as previously described (Gong et al., 2016). Briefly, ASPC-1 cells (3 × 10^4^ cells/well) were seeded into 24-well plates in triplicate and allowed to attach for 24 h. The sequences (−28∼−54) of the wild-type (WT) and mutant ASPC-1 cells were WT: TATACCCTATGGGCCAAGGGTGTGGC and mutant (MUT): CGCCTTTACTGGGAACCGGGAGCAAA. The indicated plasmids and 1.5 ng pRL-TK Renilla plasmid were transfected using Lipofectamine 3000 Reagent (Thermo Fisher Scientific, Waltham, MA, United States, cat. no. L3000008). Forty-eight hours posttransfection, luciferase and Renilla signals were assessed using a Dual Luciferase Reporter Assay Kit (Promega, cat. no. E1980) according to the manufacturer’s instructions as previously described (Hahn et al., 2002).

### Statistical Analysis

The data are presented as means ± SD. SPSS 18.0 software was used to perform statistical analyses, and graphs were generated using GraphPad Prism 8.0 (GraphPad Software, San Diego, CA, United States). Differences were examined using Student *t*-test or one-way analysis of variance. *p* < 0.05 was considered statistically significant.

## Results

### Comprehensive Analysis of the Clinical Significance of E3 Ligases in Pancreatic Cancer

**FIGURE 1:**
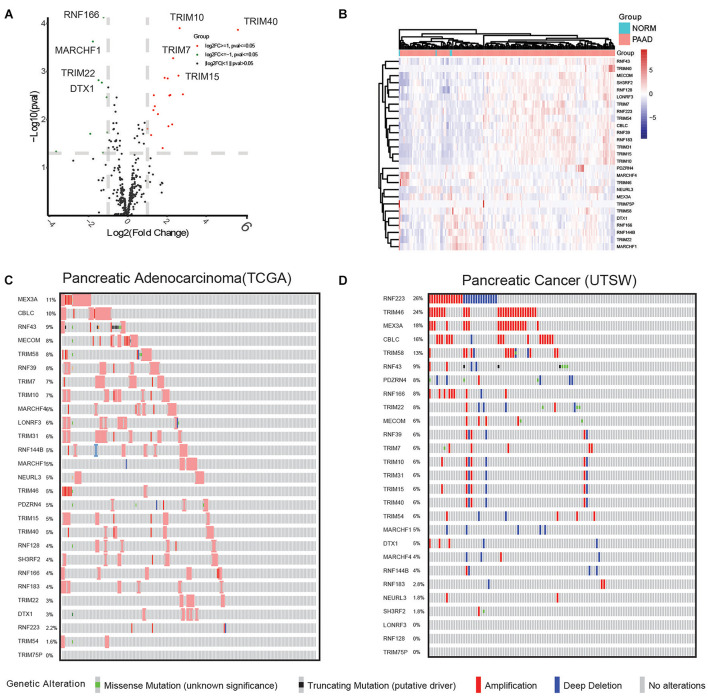
Comprehensive analysis reveals 27 differentially expressed E3 ubiquitin ligase genes and their GAs in the TCGA pancreatic cancer (PAAD) dataset. **(A)** Differential analysis of E3 ubiquitin ligases identified differentially expressed genes, shown in a volcano plot. Upregulated genes are shown as red dots, downregulated genes are shown as green dots, and genes with no significant differential expression are shown as gray dots. **(B)** Supervised hierarchical clustering of the DEGs in pancreatic cancer and normal samples. **(C)** Genetic alterations in pancreatic cancer samples included in the TCGA PAAD dataset. **(D)** Genetic alterations in pancreatic cancer samples included in the UTSW dataset.

To examine the expression differences in the E3 ligases in PC, 377 genes were applied for statistical analysis. By applying the cutoff criteria of fold change ≥ 2 or ≤ 0.5 and *p* < 0.05, 27 DEGs were acquired, including RNF166, MARCHF1, TRIM10, TRIM7, and so on (Figure 1A), and their expression in cancer and normal samples is shown in Figure 1B. Then, we examined their GAs in PC studies. As shown in Figure 1C, MEX3A exhibited the highest alteration frequency of 11%, followed by CBLC (10%), RNF43 (9%), MECOM (8%), TRIM58 (8%), and RNF39 (8%), mostly comprising amplification events. In another study (UTSW) by Witkiewicz et al. (2015), GA events displayed a comparatively different pattern. As shown in Figure 1D, RNF223 (26%), TRIM46 (24%), MEX3A (18%), CBLC (16%), TRIM58 (13%), and RNF43 (9%) showed the highest alteration frequencies. Notably, high occurrence of amplification events in MEX3A, CBLC, and MECOM and truncating mutations in RNF43 were observed in both datasets. In addition, RNF223 showed a distinct GA pattern in the two datasets, with only 2.2% in TCGA and 26% GA events (amplification and deep deletion events combined) in UTSW.

**FIGURE 2:**
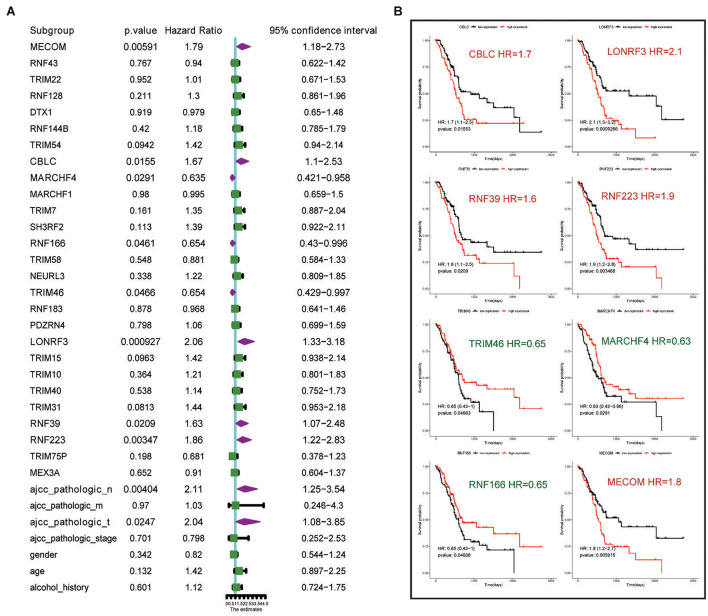
Univariate analysis highlights eight genes and clinical parameters with overall prognostic significance. **(A)** Univariate analysis of the 27 DEGs with prognostic significance in the TCGA PAAD dataset. **(B)** Kaplan–Meier survival analysis showing the HR of eight genes’ expression with OS rate in TCGA PAAD dataset. Favorable and unfavorable markers are labeled in green and red, respectively.

**FIGURE 3:**
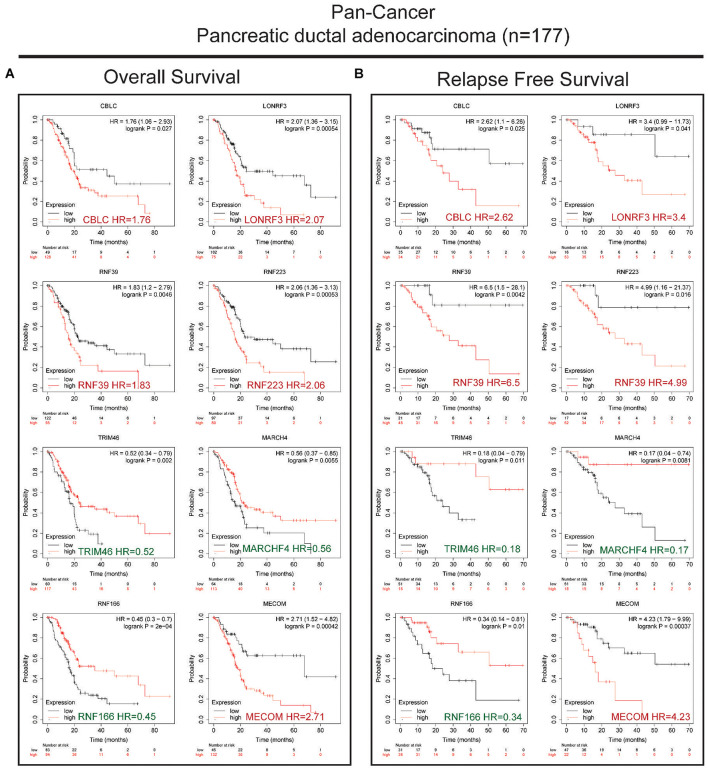
Validation of the OS and RFS rates using the PAAD dataset in a pan-cancer study. **(A)** Kaplan–Meier survival analysis showing the HR of eight genes’ expression with OS rate in the pan-cancer PAAD dataset. **(B)** Kaplan–Meier survival analysis showing the HR of eight genes’ expression with RFS rate in the pan-cancer PAAD dataset. Favorable and unfavorable markers were labeled in green and red, respectively.

Next, we conducted univariate survival analysis using the 27 DEGs and clinicopathological parameters of the TCGA dataset. As shown in Figure 2A, eight DEGs (MECOM, CBLC, MARCHF4, RNF166, TRIM46, LONRF3, RNF39, and RNF223) and two clinical parameters (pathological N stage and T stage) showed prognostic significance. The Kaplan–Meier survival plot of the eight DEGs is shown in Figure 2B. Of the eight genes, five genes, CBLC (HR = 1.7, *p* = 0.015), LONRF3 (HR = 2.1, *p* = 0.00092), RNF39 (HR = 1.6, *p* = 0.02), MECOM (HR = 1.8, *p* = 0.0059), and RNF223 (HR = 1.9, *p* = 0.0034), exhibited unfavorable prognostic significance, whereas TRIM46 (HR = 0.65, *p* = 0.046), MARCHF4 (HR = 0.63, *p* = 0.029), and RNF166 (HR = 0.65, *p* = 0.046) conveyed favorable survival significance. Finally, using the pan-cancer dataset, overall survival (OS) and relapse-free survival (RFS) analyses were conducted using the online tool Kaplan–Meier plotter. Consistent with the results in Figure 3, all eight genes showed prognostic significance, including the RFS data. Taken together, we acquired the expression and GA data of 27 DEGs, among which eight genes exhibited potential performance as prognostic indicators in PC.

### Clinical and Functional Investigation of RNF223 in Pancreatic Cancer

**FIGURE 4:**
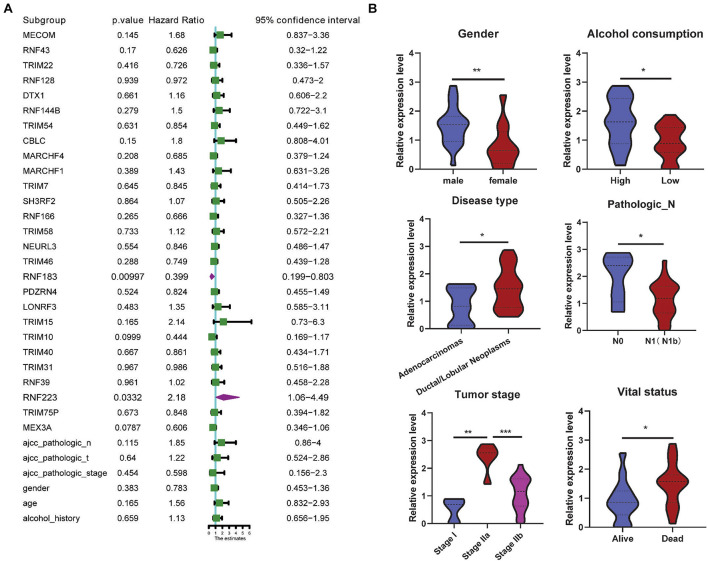
RNF223 serves as an independent prognostic marker and correlates with clinical parameters. **(A)** Multivariate analysis of the 27 DEGs with prognostic significance in the TCGA PAAD dataset. **(B)** Expression analysis of RNF23 with clinical parameters; *p < 0.05, **p < 0.01, ***p < 0.001.

Next, a multivariate analysis was conducted to analyze the prognostic significance of the 27 DEGs and the OS rate, and as a result, only RNF223 (among the eight prognostic genes) displayed prognostic significance, indicating that RNF223 may serve as an independent prognostic marker in PC (Figure 4A). Then, we compared the expression of RNF223 in groups of clinical parameters and as shown in Figure 4B, RNF223 in males (gender), high alcohol consumption, ductal/lobular neoplasms (disease type), pathological stage N0, tumor stage IIa, and vital status (dead) groups exhibited significantly higher expression compared to the other groups.

**FIGURE 5:**
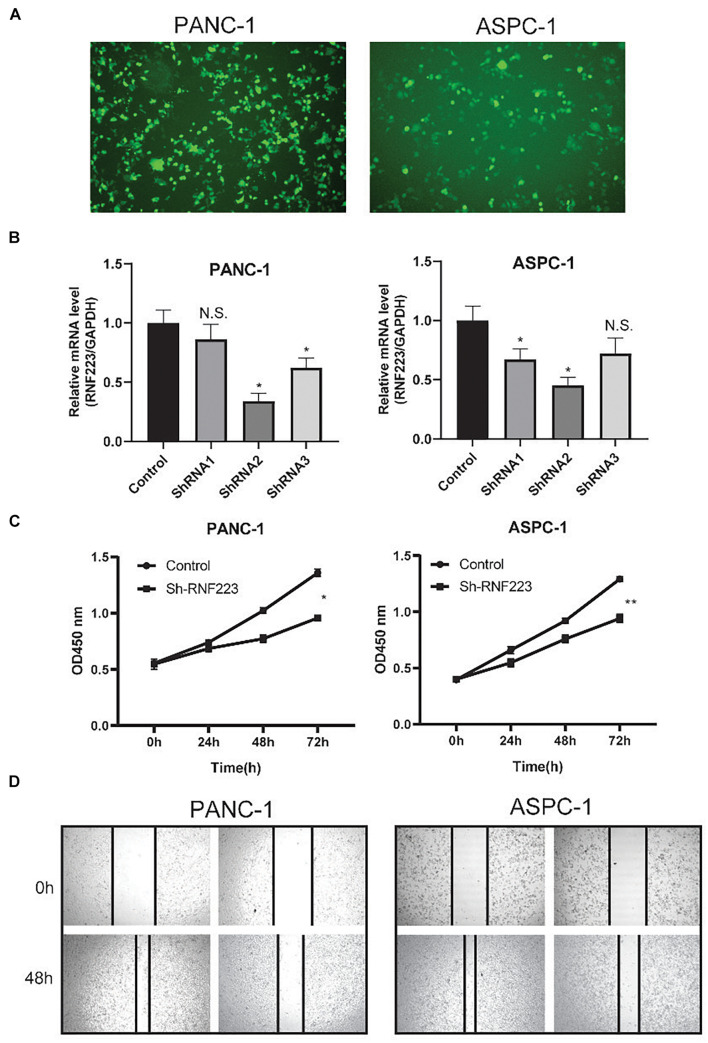
Functional investigation of RNF223 silencing in pancreatic cancer PANC-1 and ASPC-1 cell lines. **(A)** Representative cell morphology of transfection efficiency of RNF223 shRNAs in pancreatic cancer cells. **(B)** The silencing efficacy of three shRNAs was examined using qRT-PCR in pancreatic cancer PANC-1 and ASPC-1 cell lines. **(C)** The CCK8 assay was conducted to analyze the effect of RNF223 knockdown on the proliferation capability of pancreatic cancer PANC-1 and ASPC-1 cell lines. **(D)** The wound healing assay was conducted to analyze the effect of RNF223 knockdown on the migration capability of pancreatic cancer PANC-1 and ASPC-1 cell lines. All assays were conducted using three replicates and *p < 0.05, **p < 0.01.

Next, we focused on RNF223 and explored the function of RNF223 silencing on PC cell line phenotypes (Figure 5A). After RNF223-targeting shRNA transfection, the RNF223 silencing efficiency was examined using qRT-PCR, and because shRNA2 exerted consistently higher (>50%) knockdown efficiency, we used shRNA2 as the subsequent shRNA (shRNF223) (Figure 5B). Then, CCK8 and wound healing assays were applied to study the impact of RNF223 silencing on the proliferation and migration capacity of ASPC-1 and PANC-1 cells. As shown in Figure 5C, RNF223 knockdown significantly reduced the cell number in both cell lines, indicating that RNF223 may promote PC growth. In addition, the wounds of shRNF223-transfected ASPC-1 and PANC-1 cell lines demonstrated reduced migration distance compared to the control group, indicating that RNF223 knockdown decreases the migration ability in both cell lines (Figure 5D). In summary, the above results revealed that RNF223 may represent an independent prognostic marker that promotes PC growth and migration.

### The Molecular Mechanism of RNF223-Affected Pathways and Targets

**FIGURE 6:**
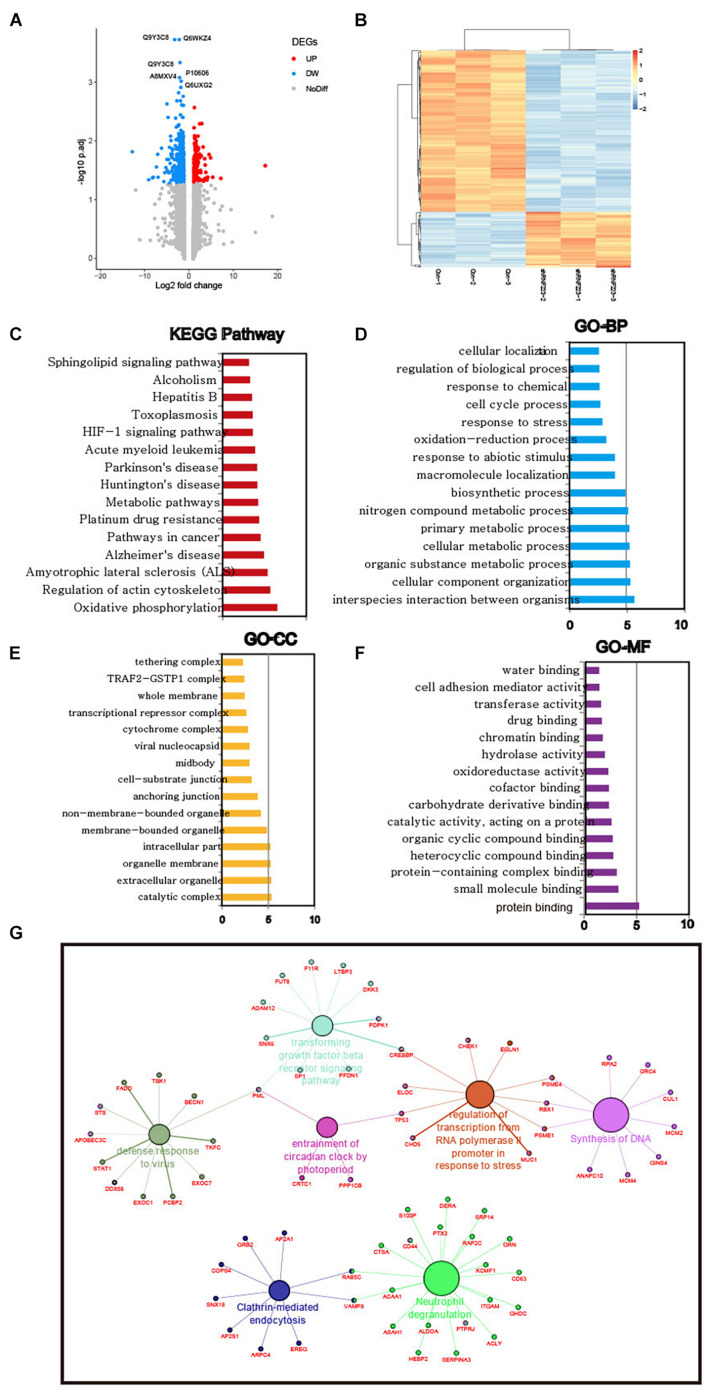
Quantitative proteomics analysis reveals pathways and BPs in the RNF223-silenced ASPC-1 cell line. **(A)** Analysis of DEGs in RNF223 knockdown ASPC-1 cells is shown using a volcano plot. Upregulated genes are shown as red dots, downregulated genes are shown as blue dots, and genes with no significant differential expression are shown as gray dots. **(B)** Supervised hierarchical clustering of the DEGs in RNF223 knockdown ASPC-1 cells. **(C)** The top 15 significantly enriched KEGG pathways of the DEGs in RNF223 knockdown ASPC-1 cells. **(D)** The top 15 significantly enriched Gene Ontology BPs of the DEGs in RNF223 knockdown ASPC-1 cells. **(E)** The top 15 significantly enriched Gene Ontology CCs of the DEGs in RNF223 knockdown ASPC-1 cells. **(F)** The top 15 significantly enriched Gene Ontology MFs of the DEGs in RNF223 knockdown ASPC-1 cells. **(G)** Functional network of the DEGs in pathways and BPs.

Then, the downstream mechanism of RNF223 in PC was investigated using quantitative proteomics in RNF223 knockdown and control ASPC-1 cells. After protein quantification, all 885 DEPs were acquired (Figure 6A), and their expression is shown in Figure 6B. Then, the functions of these DEPs were annotated, and their enriched pathways and functions were identified using KEGG pathways and GO databases. Based on the enrichment score (−log10 *p*-value), the top 15 enriched pathways and GO biological processes (BPs), cellular components (CCs), and molecular functions (MFs) are shown in Figures 6C–F. The most enriched pathways were oxidative phosphorylation, and other items, such as regulation of cytoskeleton, pathways in cancer, metabolism pathways, and HIG1α signaling pathways, were also enriched. For GO-BPs, metabolism-related BPs were also enriched, such as cellular metabolic, primary metabolic, and nitrogen compound metabolic processes. In addition, the cell cycle process was also significantly enriched. For the GO-CC result, catalytic complex was the top enriched component, supporting the pathway and BPs of enrichment of metabolism-related factors. As expected, the GO-MM category identified enriched protein binding as the most significant item, consistent with the biochemical role of RNF223 as an E3 ligase. Finally, a functional network was created of RNF223 targets, and genes in the DNA synthesis and transforming growth factor β signaling pathways are shown. In summary, we identified potential protein targets and metabolism-related pathways of RNF223 in PC (Figure 6G).

### RNF223 Was Transactivated by Kruppel-Like Factor 4 in Pancreatic Cancer

**FIGURE 7:**
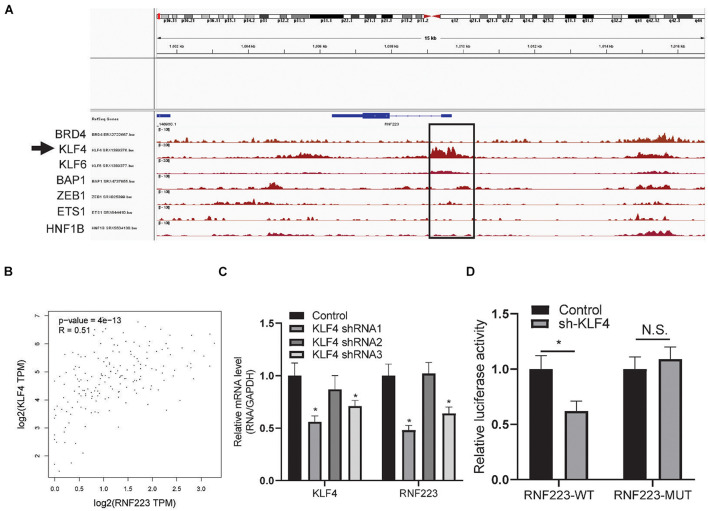
RNF223 is transcriptionally activated by KLF4 in pancreatic cancer. **(A)** Binding peaks of transcription factors in pancreatic cancer tissue and cell lines; arrow indicates that KLF4 shows remarkable binding affinity at the promoter of RNF223. **(B)** Expression correlation of KLF4 with RNF223 in the TCGA PAAD dataset. **(C)** Relative expression of KLF4 and RNF223 in KLF4 knockdown shRNA-transfected ASPC-1 cell lines. **(D)** Luciferase assay showing the binding affinity of KLF4 in RNF223 promoter WT and mutated (MUT) cells. All assays were conducted using three replicates and *p < 0.05.

Finally, the mechanism underlying the elevated expression of RNF223 was explored. As the genetic alteration frequency of RNF223 was not remarkably high in PC, we speculated that transcription factors may contribute to this process. First, we downloaded PC ChIP sequencing data from the recently published online tool ChIP-Atlas. As shown in Figure 7A, KLF4 exhibited a prominent peak in the promoter region of RNF223 DNA. In addition, coexpression analysis of KLF4 with RNF223 revealed a strong coefficient (*R* = 0.51) in TCGA PAAD datasets (Figure 7B). Then, to validate the role of KLF4 on the mRNA expression of RNF223, qRT-PCR was performed to examine the expression of RNF223 in the KLF4 knockdown cell line ASPC-1. As shown in Figure 7C, RNF223 exhibited significantly decreased expression in KLF4-silenced cells, indicating that KLF4 may upregulate RNF223 expression in ASPC-1 cells. Finally, a luciferase assay was conducted to validate the above results. As shown in Figure 7D, KLF4 silencing remarkably decreased luciferase intensity in the RNF223 WT group, whereas no significant difference was observed in the RNF223 MUT group. Altogether, these data show that KLF4 contributes to the increased expression of RNF233 in PC.

## Discussion

Mounting evidence indicates that E3 ubiquitin ligases play important roles in cancer onset, progression, and treatment response and serve as prognostic makers in cancer (Senft et al., 2018). Both genetic and epigenetic alterations account for the dysregulation of E3s in cancer (Qi and Ronai, 2015). Consequently, the stability and/or activity of E3 substrates are also altered, leading to downregulation of tumor-suppressor activities and upregulation of oncogenic activities (Senft et al., 2018; Fujita et al., 2019). Targeting E3 ligases has been previously proposed as a novel cancer therapeutic strategy (Micel et al., 2013; Sharp et al., 2021). A better understanding of the mechanisms underlying E3 regulation and function in tumorigenesis is expected to reveal novel prognostic markers and to enable the development of the next generation of anticancer therapies (Kumari et al., 2017; Wang et al., 2017; Khan et al., 2020). Here, by analyzing the clinical significance of E3 ligases in PC, we first identified 27 DEGs, of which eight DEGs showed prognostic performance. To the best of our knowledge, this is the first comprehensive study of E3 ligases in PC and provides an overall map of these E3 ligases in PC.

Among the eight prognostic markers, most have been reported in cancer. For instance, MDS1 and EVI1 complex loci (MECOM) interact with PAX8 and drive oncogenic functions in ovarian cancer (Bleu et al., 2021). Cbl Proto-Oncogene C (CBLC) was demonstrated to enhance epidermal growth factor receptor dysregulation and signaling in lung adenocarcinoma (Hong et al., 2018). MARCHF4, previously known as MARCH4, was identified as a potential therapeutic target in cutaneous squamous cell carcinoma (McHugh et al., 2020). The chimeric RNAs generated from tripartite motif containing 46 (TRIM46) with MUC1 and KRTCAP2 have been clinically implicated in high-grade serous ovarian carcinoma (Kannan et al., 2015). In PC, MECOM was shown to be a critical regulator that suppresses acinar cell death by permitting cellular dedifferentiation (Backx et al., 2021), but there are limited studies regarding the other seven genes in pancreatic cancer. Identification of these eight genes provides an alternative option for prognostic prediction in pancreatic cancer patients.

Subsequently, we identified RNF223 as an independent prognostic marker in pancreatic cancer, and further functional assays revealed that RNF223 may play an oncogenic role in pancreatic cancer progression. As a member of the ring finger proteins, most studies have focused on RNF43. RNF43 mutation might cause downregulation of the expression of ring finger protein 43 and associate synergistically with GNAS mutations during the development of PC (Sakamoto et al., 2015). In addition, mutational inactivation of RNF43 confers Wnt dependency and could be used as a predictive biomarker for the clinical development of Wnt inhibitors in PC (Jiang et al., 2013). For RNF223, mutation sites have been related to age, International Federation of Gynecology and Obstetrics stage, and histology in sporadic and Lynch syndrome–associated endometrial cancer (Sun et al., 2021). In addition, RNF223 was reported to serve as a prognostic marker for uterine sarcoma (Zhou et al., 2019). To date, no studies of RNF223 have been reported in PC. Moreover, we are conducting additional assays to reveal the role of RNF223 in PC, including the clinical significance of RNF223 in our collected PC samples, *in vivo* xenograft animal assays, and immunoprecipitation-coupled MS to identify targets of RNF223 in PC. In addition, as E3 ligases have been shown to function in both ubiquitin–proteasome-dependent and ubiquitin–proteasome-independent diseases (Cadena et al., 2019; Weinelt and van Wijk, 2021), the specific mechanism of RNF223 in PC remains to be uncovered in the future.

Finally, utilizing the ChIP data in ChIP-Atlas and further validation assays, such as luciferase assays, we identified KLF4 to be a hub regulon of RNF223 in PC. KLF4 has been widely reported as an oncogene in multiple cancer types (Rowland and Peeper, 2006; Hu et al., 2015; Hsieh et al., 2017; Murgai et al., 2017), including lung cancer (Yu et al., 2016), ovarian cancer (Zhang et al., 2019), esophageal squamous cell cancer (Tetreault et al., 2010), gastric cancer (Li et al., 2012), colorectal cancer (Li et al., 2011; Gamper et al., 2012), and leukemia (Faber et al., 2013; Li et al., 2015; Seipel et al., 2016; Park et al., 2019). In PC, KLF4 was demonstrated to contribute to carcinogenesis and progression by inducing acinar-to-ductal reprogramming (Wei et al., 2016), as well as the LDHA signaling pathway and aerobic glycolysis (Shi et al., 2014) and MSI2 signaling pathway–mediated cell growth (Guo et al., 2017). Moreover, increased expression of KLF4 is attributed to hypomethylation-mediated by DNA methyltransferase 1 (Xie et al., 2017). In this study, we first validated RNF223 as a novel target of KLF4, and the primary enriched metabolic pathways of RNF223 also corresponded to the function of KLF4 as a regulator of glycolysis.

## Conclusion

This study comprehensively analyzed the expression difference in E3 ligases and identified eight prognostic markers among 27 DEGs. In addition, functional assays of RNA silencing revealed RNF223 as a tumor-promoting gene that may regulate cancer cell metabolism. Finally, the elevated expression of RNF223 was attributed to KLF4-mediated transcriptional activation. This study broadens our knowledge of E3 ubiquitin ligases and signal transduction and provides novel markers and therapeutic targets for PC.

## Data Availability Statement

The datasets presented in this study can be found in online repositories. The names of the repository/repositories and accession number(s) can be found below: iProX; PXD028446.

## Ethics Statement

The study protocol was reviewed and approved by the Zhujiang Hospital Institutional Review Board.

## Author Contributions

CZ, YG, and LF were responsible for the conception and design and study supervision. JW, JZ, JD, and LH were responsible for the development of the methodology, analysis, and experiments. CZ, LF, JW, JZ, CF, HL, and XX performed the statistical and bioinformatic analysis. All authors read and approved the final manuscript.

## Conflict of Interest

The authors declare that the research was conducted in the absence of any commercial or financial relationships that could be construed as a potential conflict of interest.

## Publisher’s Note

All claims expressed in this article are solely those of the authors and do not necessarily represent those of their affiliated organizations, or those of the publisher, the editors and the reviewers. Any product that may be evaluated in this article, or claim that may be made by its manufacturer, is not guaranteed or endorsed by the publisher.

## Footnotes

1
https://portal.gdc.cancer.gov/

2
https://hpcwebapps.cit.nih.gov/ESBL/Database/E3-ligases/

3
https://maayanlab.cloud/Enrichr/

4
http://cbioportal.org

5
www.kmplot.com

6
http://chip-atlas.org/

## Supplementary Material

Supplementary Figure 1 The flowchart of this study.

## Abbreviations

TCGA: The Cancer Genome Atlas
RNF: RING finger
DEGs: differentially expressed genes
GO: Gene Ontology
CCK8: cell counting kit-8
HR: hazard ratio
DIA: data-independent acquisition
FDR: false discovery rate
TSS: transcription start sites
GA: genomic alterations
OS: overall survival
RFS: relapse-free survival
DEPs: differentially expressed proteins
BP: biological processes
CC: cellular components
MF: molecular functions
WT: wild type
MUT: mutant
MECOM: MDS1 and EVI1 complex locus
TRIM46: tripartite motif containing 46
KLF4: Kruppel-like factor 4.
