# Supplementary figures and images for: Comprehensive Analysis of E3 Ubiquitin Ligases Reveals Ring Finger Protein 223 as a Novel Oncogene Activated by KLF4 in Pancreatic Cancer
Source: Front Cell Dev Biol. 2021 Oct 14;9:738709. doi: 10.3389/fcell.2021.738709 (PMC8551701; doi:10.3389/fcell.2021.738709)

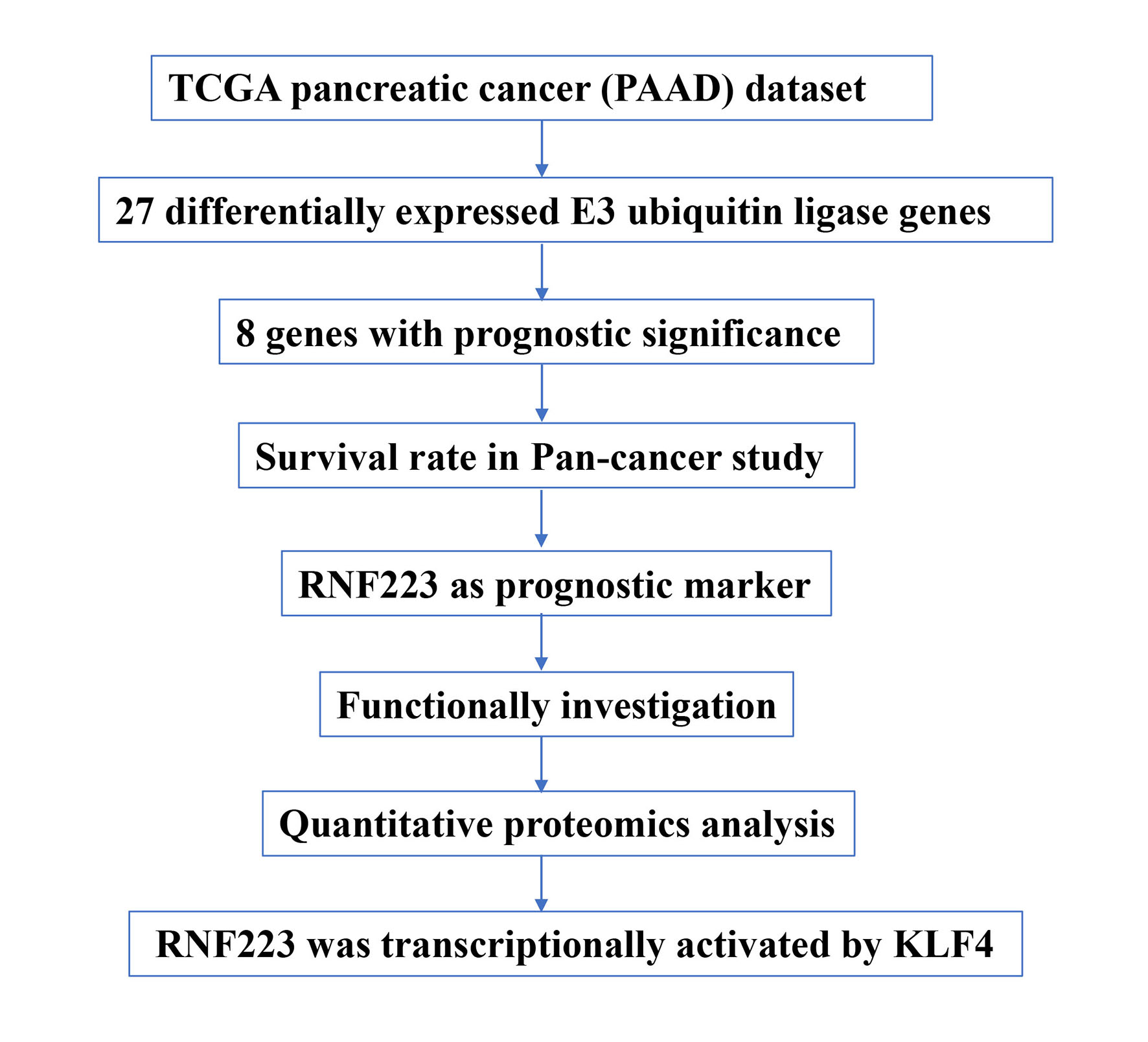

Supplement: Supplementary Figure 1 — The flowchart of this study. [file Image_1.JPEG]
